# Definitions and assessments of physical literacy among children and youth: a scoping review

**DOI:** 10.1186/s12889-023-16680-x

**Published:** 2023-09-07

**Authors:** Martin Grauduszus, Stefanie Wessely, Marlen Klaudius, Christine Joisten

**Affiliations:** Department for Physical Activity in Public Health, Institute of Movement and Neurosciences, German Sport University Cologne, Am Sportpark Müngersdorf 6, Cologne, 50933 Germany

**Keywords:** Physical literacy, Assessment, Youth, Children, Holistic

## Abstract

**Background:**

Despite the recognised health benefits of physical activity, the physical activity levels of children and adolescents continue to decline. The concept of physical literacy (PL) is a promising holistic approach to physical activity promotion that addresses affective and cognitive domains in addition to physical and motor domains. In Germany, however, no uniform or widely used method exists for assessing PL in children. This research was conducted to compile information on international PL assessment systems for children and adolescents (up to 18 years of age), including their underlying definitions, structural designs and development processes, for the purpose of developing such a tool in Germany.

**Methods:**

A scoping review was conducted using PubMed, Web of Science and SPORTDiscus database entries. The initial search was conducted in July 2022, with a follow-up search performed in May 2023. Articles that operationalised the construct of PL and at least two of the three domains were identified and included. The procedure and assessment tools used to evaluate the individual domains and the overall PL construct were extracted from all selected articles.

**Results:**

A total of 882 articles were identified; five were added after a manual search. After duplicates were removed, 563 articles were screened by title and abstract, and 40 articles met the inclusion criteria and were included in the review. In a review of these articles, 23 different assessment procedures were identified. Eight assessment procedures included PL as a superordinate construct. Twenty-two of the 23 procedures assessed the affective and physical domains, only 14 assessed the cognitive domain.

**Conclusion:**

Approximately half of the identified PL assessment systems addressed all three domains. Motor performance was most frequently integrated into the test procedures. Future developments in Germany should integrate all domains in the assessment to produce a holistic conceptualisation as the basis for appropriate funding.

## Background

Physical activity and exercise play central roles in the healthy physical, psychosocial, cognitive, and emotional development of children and adolescents [1–3]. Despite this knowledge, even before the COVID-19 pandemic, only 15% of adolescents aged 11–17 years worldwide engaged in the 60 min of physical activity per day recommended by the World Health Organization (WHO) [4]. In Germany, according to the second KIGGs Wave cross-sectional study, similarly low physical activity levels were observed in children aged 7–10 years: only 23% of girls and 30% of boys reached the recommended physical activity levels [5]. During the COVID-19 pandemic, children’s physical activity time further decreased between 10.8 min/day and 91 min/day [6].

Due to the potential negative consequences of insufficient physical activity, including excessive weight gain, obesity, and motor deficits [7], the WHO Global Action Plan on Physical Activity 2018–2030 called for reducing the global prevalence of physical inactivity by 15% by 2030 [8]. To this end, programmes promoting individual physical activity behaviours should be developed and expanded. Thus far, however, despite a multitude of actions taken by various institutions and schools [9–11], a reversal in the downward trend has not been achieved [12]. Comprehensive strategies may thus be needed that target daily life and living environment and address additional factors such as children’s and adolescents’ intrinsic motivation and self-efficacy for initiating and maintaining an active or healthy lifestyle [13, 14]. Such considerations can already be found in process models of behavioural change pertaining to health-related behaviours [15]. Knowledge and understanding of the effects and impacts of physical activity also support individuals’ sense of responsibility for their own (health) behaviours [16].

One possible theoretical basis for such interventions, which recognise this broader understanding of exercise and the promotion of exercise, is the concept of physical literacy (PL) [17]**.** Margaret Whitehead proposed this term to describe participation and physical activity behaviour with a philosophical underpinning. According to Whitehead, the human being exists as a unity of body and mind (monism) and is the result of accumulated experiences in the world (existentialism), which form the basis for one’s process of perception (phenomenology) [18]. Movement experiences thus frame future behaviour and shape the domains of PL, which include the cognitive domain (knowledge and understanding of the physical and psychological effects of sport and exercise), the affective domain (regulation of motivation, movement-related self-efficacy, and self-confidence), and the physical domain (movement, sports participation, motor skills, and fundamental movement skills). These domains exist not in isolation but instead in relation to each other. For example, motor skills (physical domain) are considered a prerequisite for participation in a country’s physical activity and sports culture [19], and they also affect self-efficacy and intrinsic motivation (affective domain). Additionally, the interrelated domains, as PL, may form the basis for a lifelong process [17].

Various culturally adapted definitions and (depending on the field of application) corresponding assessment systems have been developed from this theoretical framework [20–23]. Among the most well-known assessment instruments is the Canadian Assessment of Physical Literacy (CAPL), which is employed internationally and has been translated and culturally adapted for use in several countries [24–29].

Currently no model or procedure for assessing PL is available for any age group in Germany. Before effective physical activity support measures for children and adolescents can be developed and compared, an adequate operationalisation of the domains or construct and a clear and uniform definition are needed [30]. For this purpose, a scoping review was conducted to compile information on existing PL assessment systems for children and adolescents, including their underlying definitions, structural designs, and development and evaluation processes, and facilitate both a review of common approaches and the identification and discussion of the most suitable methods for measuring PL. The results of this study can therefore form a cornerstone of a German system for PL assessment on which future PL developments can build.

## Methods

A scoping review was conducted according to the guidelines of the Joanna Briggs Institute [31] and the framework employed by Arksey and O’Malley [32].

### Search and screening process

An initial systematic literature search was conducted in July 2022 and followed by an updated search in May 2023 using three electronic sports science and medical databases: (i) MEDLINE (via PubMed), (ii) Web of Science, and (iii) SPORTDiscus. The literature search results were manually checked for duplicate publications. The search strategy combined the term ‘physical literacy’ and the target group of children and adolescents (‘physical literacy’ AND (‘children’ OR ‘childhood’ OR ‘youth’ OR ‘adolescent’)).

Two independent reviewers (MG and SW) screened the identified articles for the specified inclusion criteria. The screening process was carried out using the program Rayyan [33] and was divided into two successive stages: (1) title and abstract screening and (2) full-text screening. Duplicates were removed. Any disagreements were discussed after each stage. If a consensus could not be reached, a third researcher (CJ) was consulted. Additionally, a search for further relevant articles was conducted by exploring the reference lists of the included studies. The inclusion criteria were specified using the PCC (participants, concept, context) scheme:

- Participants: The target group of the articles had to include children and adolescents up to 18 years of age. Articles were excluded if the target group included children with specific pre-existing conditions, such as obesity.
- Concept: Only articles that focused on or operationalised the construct of PL were included. In addition, validation studies of PL assessment instruments were included. At least two of the three constituent PL domains (cognitive, affective, physical) had to be covered in the assessment procedure. The cognitive domain was understood to be knowledge and understanding of changes to the body and psyche due to movement. The affective domain referred to the areas of motivation, self-efficacy, and self-confidence. The physical domain was related to motor skills, movement behaviour and fundamental movement skills.
- Context: All institutions for children and young people, such as kindergartens, schools, youth centres and community projects, were included as settings for the PL assessment systems. All nationalities were included. Assessment systems conducted at universities were excluded.

The exclusion criteria included contributions to conferences, scientific posters, and non-English or non-German articles. Conference papers were excluded due to their tendency to present the applied methodology with limited detail and incompleteness. If full-text access to a publication was unavailable, the author was contacted and asked to send the full text.

### Data items and charting

A standardised extraction protocol for summarising relevant variables was developed for this review. In accordance with the Preferred Reporting Items for Systematic Reviews and Meta-Analyses (PRISMA) checklist for scoping reviews, the reviewers first checked five articles for completeness and applicability [34]. The extraction protocol was then adapted based on this pilot review and included the following elements:

- Name of the first author, year of publication, and country of publication;
- The target group of the assessment tool;
- The underlying PL definition;
- Evaluation that the articles included an assessment of PL as a superordinate construct;
- The terminology used for the domains;
- The name and characteristics of the instrument used to evaluate each domain;
- The assessment of each domain.

## Results

### Literature search and study characteristics

**Fig. 1:**
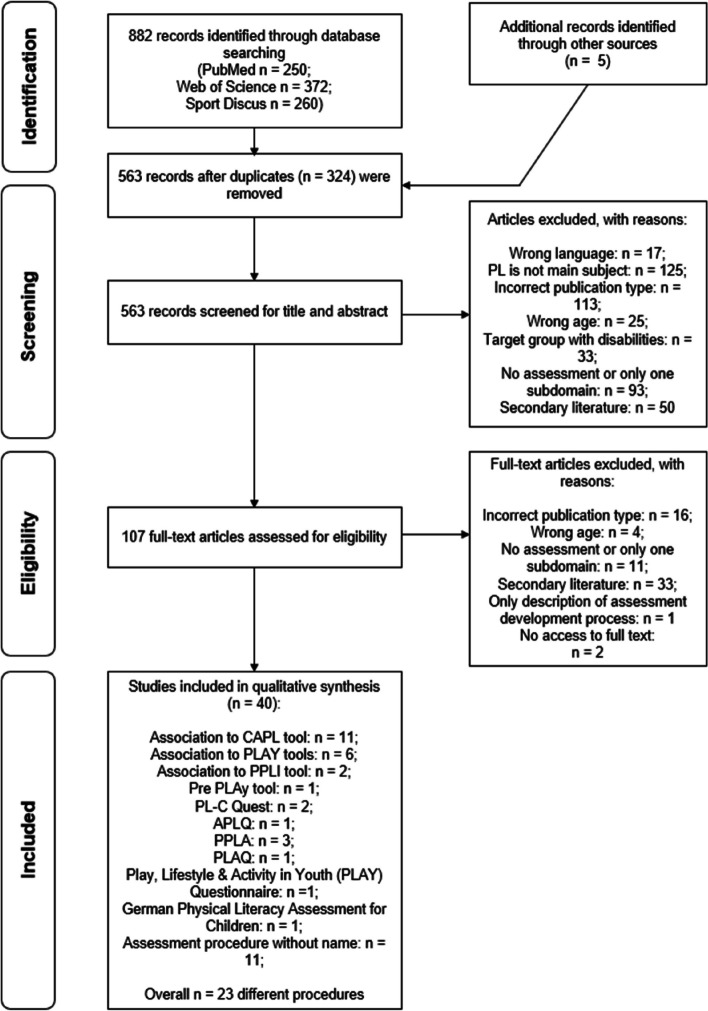
PRISMA flow diagram showing the process of study selection. *Abbreviations*: *CAPL* Canadian Assessment of Physical Literacy, *PL* Physical Literacy, *PLAY* Physical Literacy Assessment for Youth, *PPLI* Perceived Physical Literacy Instrument, *Pre PLAy* Preschool Physical Literacy Assessment Tool, *PL-C Quest* Physical Literacy in Children Questionnaire, *APLQ* Adolescent Physical Literacy Questionnaire, *PPLA* Portuguese Physical Literacy Assessment, *PLAQ* physical literacy self-assessment questionnaire

The literature search identified 882 articles (Fig. 1). Five additional sources that fulfilled the inclusion criteria were added manually. After duplicates were removed, the titles and abstracts of 563 articles were checked for inclusion criteria. In the next step, 107 full texts were processed and checked for suitability, from which 40 articles met all criteria and were included in data extraction. Twenty-three PL assessment procedures were described in the 40 articles. Eleven articles were related to the CAPL and its further development (Canadian Assessment of Physical Literacy Second Edition, CAPL-2; Canadian Assessment of Physical Literacy in grades 7–9, CAPL 789) by the Healthy Active Living and Obesity Research Group [24–29, 35–39]. Seven sources were identified pertaining to the Physical Literacy Assessments for Youth (PLAY) and the Preschool Physical Literacy Assessment Tool (Pre PLAy) [40–46]. Two articles described the Perceived Physical Literacy Instrument (PPLI) [47, 48]. Three articles described the Portuguese Physical Literacy Assessment (PPLA) consisting of the Portuguese Physical Literacy Assessment-Questionnaire (PPLA-Q) and the Portuguese Physical Literacy Assessment-Observation (PPLA-O) [49–51]. The development of the Physical Literacy in Children Questionnaire (PL-C Quest) was described in two articles [52, 53]. One article each dealt with the Adolescent Physical Literacy Questionnaire (APLQ) [54], the Physical Literacy Self-Assessment Questionnaire (PLAQ) [55], the Play, Lifestyle & Activity in Youth Questionnaire [56], and the German Physical Literacy Assessment for Children [57]. Eleven other methods focused on individual PL domains and were not validated with respect to use as a PL tool [58–68].

### General procedure for the development of PL test instruments

The test procedures identified in the literature can be found in Table 1. In general, the objective and target group(s) of the instrument were determined first, followed by a determination of the underlying definition of PL based on literature research [42, 46, 49, 54, 57, 58, 60–68], Delphi procedures [37], and interviews with practitioners and experts [48, 49, 52, 54, 55]. In a subsequent step, either survey methods of the domains were developed or existing methods were used. These methods varied between observational questionnaires, validated or newly developed questionnaires, and motor skills tests. The choice of test methods depended on the underlying definition of the respective domain (see Tables 2, 3 and 4).

**Table 1:** Underlying physical literacy definition of the assessment conception (*n* = 23)

Author(s) and year	Name of the assessment tool	Country of origin	Age of target group and study population	Physical literacy definition or explanation	Further literature that assesses test quality
Mohammadzadeh et al. (2021) [54]	Adolescent Physical Literacy Questionnaire (APLQ)	Iran	Children aged 11–18 years old	Following Whitehead's and International Physical Literacy Association’s (IPLA) definition: ‘physical literacy emphasises motivation, self-confidence, and physical competence, noting that physical literacy is designed to help individuals take responsibility for being active’ [17, 69]	[54]
Longmuir et al. (2015) [24]	Canadian Assessment of Physical Literacy (CAPL)	Canada	Children aged 8–12 years old	‘There are four interconnected and essential elements of physical literacy: motivation and confidence (affective domain), physical competence (physical domain), knowledge and understanding (cognitive domain), and engagement in physical activities for life (behavioural domain).‘	[24]
Longmuir et al. (2018) [38]	Canadian Assessment of Physical Literacy 2 (CAPL-2); further development of CAPL	Canada	Children aged 8–12 years old	‘Motivation, confidence, physical competence, knowledge and understanding to value and take responsibility for engagement in physical activities for life’. This definition is adopted from the IPLA [69]	[25–29]
Blanchard et al. (2020) [39]	Canadian Assessment of Physical Literacy in grades 7–9 (CAPL 789)	Canada	Children aged 12–17 years old	‘Motivation, confidence, physical competence, knowledge and understanding to value and take responsibility for engagement in physical activities for life’. This definition is adopted from the IPLA [69]	[39]
Krenz et al. (2022) [57]	German Physical Literacy Assessment for Children	Germany	Children aged 6–12 years old	‘The motivation, confidence, physical competence, knowledge, and understanding to maintain physical activity throughout the lifecourse.’ [69]	[57]
Sum et al. (2018) [47]	Perceived Physical Literacy Instrument for adolescents (PPLI)	Hong Kong	Children aged 11–19 years old	‘Physical literacy is a specific intelligence that includes the motivation, confidence, physical competence, and knowledge and understanding to value and take responsibility for maintaining purposeful physical pursuits and activities throughout the course of one’s life.’	[47]
PLAY tools (2013) [42]	Physical Literacy Assessment for Youth (PLAY) tools	Canada	Children aged 7 years old and above	‘Physical literacy is defined as the motivation, confidence, physical competence, knowledge, and understanding to value and take responsibility for engagement in physical activity for life.’ [69]	[40, 41, 43–45]
Barnett et al. (2022) [52]	Physical Literacy in Children Questionnaire (PL-C Quest)	Australia	Children aged 4–12 years old	‘Physical literacy was characterised as the integration of physical, psychological, social and cognitive capabilities that help us live active, healthy and fulfilling lifestyles.’ [70] The Australian Physical Literacy Framework extends the IPLA definition, as it covers four domains (physical, psychological, social and cognitive) and includes 30 elements within these domains	[52, 53]
YongKang & QianQian (2022) [55]	Physical Literacy self-assessment questionnaire (PLAQ)	China	Children aged 8–12 years old	‘The motivation, confidence, physical competence, knowledge, and understanding to maintain physical activity throughout the lifecourse.’ [69]	[55]
Stracciolini et al. (2021) [56]	Play, Lifestyle & Activity in Youth Questionnaire	USA	Children aged 6–11 years old	‘A child who might be considered physically literate is not only physically competent but also makes conscious decisions to correctly control daily behavior, develops and nurtures their confidence and motivation, and has the knowledge and understanding to value and take responsibility for participation in physical activity for life.’ [69]	NA
Mota, Martins & Onofre (2021) [49]	Portuguese Physical Literacy Assessment (PPLA)	Portugal	Adolescents aged 15–18 years old	Following the Australian Physical Literacy Framework [70] and the Portuguese PE national syllabus [71] ‘The Portuguese PE national syllabus (PPES) was designed under the Crum’s socio-critical conception of PE, contemplating integrated learning in the motor, cognitive, affective and social domains, to empower students to engage in significant physical activity (PA), and actively participate in the movement culture throughout their lives’	[49–51]
Cairney et al. (2018) [46]	The Preschool Physical Literacy Assessment Tool (Pre PLAy)	Canada	Infants aged 18–49 months	‘Physical literacy encompasses the knowledge, skills, motivation, and feelings related to physical activity and movement.’	[46]
Belton et al. (2019) [59]	NA	Ireland	Children aged 12–14 years old	‘Motivation, confidence, physical competence, knowledge, and understanding to value and take responsibility for maintaining purposeful pursuits throughout the lifecourse.’ This definition is adopted from the IPLA [69]	NA
Britton et al. (2022) [60]	NA	Ireland	Children aged 9–12 years old	‘Whitehead’s definition, comprising confidence, motivation, physical competence, and knowledge and understanding.’ [17]	[60]
Brown et al. (2021) [61]	NA	Canada	Children aged 8–13 years old	‘PL has been shown to be a higher-order latent construct that encompasses, at least, motor competence, confidence, motivation and enjoyment. […] it is worthwhile to note that knowledge regarding the importance of PA also plays an influential role in this dynamic process.’	NA
Cairney et al. (2018) [62]	NA	Canada	Children aged 8–13 years old	‘(a) Competence in movement skills, (b) perceived movement competence, (c) motivation, (d) enjoyment of physical activity, and (e) self-knowledge related to health. Consistent with much of the conceptual work on PL we view physical activity behaviours, body composition, and fitness as manifestations of PL, rather than part of the construct itself.’	[62]
Demetriou et al. (2018) [63]	NA	Germany	Primary school children	‘Physical literacy represents the successful interaction of four inter-related core domains: (a) physical fitness (cardiovascular fitness, muscular strength and endurance, flexibility, and coordination); (b) fundamental motor skills (e.g., catching and throwing a ball); (c) physical activity behaviours, and (d) psycho-social/cognitive factors (attitudes, knowledge, and feelings).’	NA
George et al. (2016) [58]	NA	Canada	Children aged 6–12 years old	‘Physical literacy includes four main domains: physical activity behaviours, physical fitness, awareness/knowledge and understanding, and motor skills.’ [72]	NA
Gu et al. (2019) [64]	NA	United States of America	Children aged 8 and 9 years old	Physically literate individuals are expected to demonstrate the ‘ability to move with competence and confidence in a wide variety of physical activities in multiple environments that benefit the healthy development of the whole person’ [73]. In line with the performance-driven perspective of PL, this study examined fundamental performance indicators, including knowledge, skills and behaviours [74]	NA
Guerrero et al. (2018) [65]	NA	Canada	Children aged 8–10 years old	‘[…] motivation, confidence, physical competence, knowledge and understanding to value and engage in physical activities for life. It comprises four dynamic, interconnected domains: affective (motivation and confidence), physical (physical competence), cognitive (knowledge and understanding), and behavioural (engagement in a physically active lifestyle).’	NA
Rudd et al. (2020) [66]	NA	England	Children aged 5–6 years old	‘Physical literacy can be understood as the embodied relationship between a child’s movement competence (physical), motivation and confidence (affective), knowledge and understanding (cognitive) and their environment, which shapes movement and ongoing physical activity behaviours’ [75]	NA
Telford et al. (2021) [67]	NA	Australia	Children aged 10 and 11 years old	‘The term physical literacy defined as the motivation, confidence, physical competence, knowledge, and understanding to value and take responsibility for engaging in physical activities for life.’	NA
Yli-Piipari et al. (2021) [68]	NA	Finland	Children aged 10–12 years old	‘[…] a physically literate individual should: (a) be competent in motor skills, (b) engage in a healthy dose of daily PA, (c) demonstrate health-enhancing levels of fitness, and (d) be motivated toward and enjoy participating in regular PA.’	NA

*Abbreviations*: *APLQ* Adolescent Physical Literacy Questionnaire, *CAPL* Canadian Assessment of Physical Literacy, *CAPL-2* Canadian Assessment of Physical Literacy second edition, *CAPL 789* Canadian Assessment of Physical Literacy in grades 7–9, *IPLA* International Physical Literacy Association, *NA* Not available or data could not be extracted, *PPLI* Perceived Physical Literacy Instrument, *PLAY* Physical Literacy Assessment for Youth., *PL-C Quest* Physical Literacy in Children Questionnaire, *PLAQ* physical literacy self-assessment questionnaire, *PPLA* Portuguese Physical Literacy Assessment, *Pre PLAy* Preschool Physical Literacy Assessment Tool, *PA* Physical activity, *PL* Physical Literacy

**Table 2:** Assessment tools and scoring of the physical domain (*n* = 23)

Author(s), year and assessment name	Physical domain: terminology	Physical domain: instrument name	Physical domain: assessment procedure
Mohammadzadeh et al. (2021); APLQ [54]	Physical fitness and physical activity	Adolescent Physical Literacy Questionnaire (APLQ); Self-report questionnaire; 6 items	5-point Likert scale (1: ‘Strongly Disagree’; 5: ‘Strongly Agree’). Each item is assigned a maximum of 5 points
Longmuir et al. (2015); CAPL [24]	Physical competence: Fundamental, complex and combined movement skills	Canadian Agility and Movement Skill Assessment [76]	Time and quality of skills were assessed on an obstacle course. Overall, 28 points could be achieved: a maximum of 14 points for time based on normative data and a maximum of 14 points for quality in 7 different skills (where every skill was graded from 1 to 3 points). Scores were divided by 2.8 for a max score from 10
	Physical competence: muscular endurance	Isometric plank hold [77]	Time in seconds in isometric plank position; normative score building; max. 10 points
	Physical competence: cardiorespiratory endurance	The Progressive Aerobic Cardiovascular Endurance Run [78]	The number of lengths completed, including the first length that did not reach the line, was recorded; normative score building; max. 10 points
	Physical competence—muscular strength	Handgrip dynamometry [79]	Grip strength with maximum score in kilogram for each hand; max 17 points
	Physical competence – flexibility	Sit and reach protocol [79]	Max 17 points
	Physical competence—body composition	Standing height, body mass, waist circumference [79]	For Body Mass Index (BMI) percentile max 17 points; for waist circumference max. 17 points
Longmuir et al. (2018); CAPL-2 [38]	Physical competence: Fundamental, complex and combined movement skills	Canadian Agility and Movement Skill Assessment [76]	Time and quality of skills were assessed on an obstacle course. Overall, 28 points could be achieved: a maximum of 14 points were awarded for time based on normative data, for quality, a maximum of 14 points in 7 different skills (each skill was awarded 1–3 points). Scores were divided by 2.8 for a max score from 10
	Physical competence: aerobic endurance	The Progressive Aerobic Cardiovascular Endurance Run [78]	The number of lengths completed, including the first length that did not reach the line, were recorded; normative score building; max. 10 points
	Physical competence: muscular endurance	Isometric plank hold [77]	Time in seconds in isometric plank position; normative score building; max. 10 points
	Daily Behaviour	Pedometer	The child’s average daily step count from the pedometer assessment is awarded a maximum of 25 points (from 0 points for < 2,000 steps to 25 points for > 17,999 steps), with higher points assigned for the performance of more steps per day and 17 points awarded for achieving the recommended 12,000 steps per day
		CAPL-2 questionnaire; 1 item; self-reported number of days in the past week that they were physically active for at least 60 min per day	A maximum of 5 points for self-reported 6 or 7 days; minimum for 0–1 self-reported days in the past week
Blanchard et al. (2020); CAPL 789 [39]	Procedure matched CAPL-2	Procedure matched CAPL-2	Procedure matched CAPL-2
Krenz et al. (2022) [57]	Motor skills	Dordel-Koch Test; 3 items [80]	Product-based scoring with classification from 1 (worst rating) to 6 (highest rating) based on normative data
	Participation	German Physical Literacy Assessment for Children – Questionnaire; For children who were 6 or 7 years old the questions were answered in an interview. Older children filled in the questionnaire themselves; 3 items	6-point circle analogue scale, ranging from the smallest circle 1 (‘not at all confident’) to the biggest circle 6 (‘fully confident’)
PLAY-tools (2013) [42]	Physical development	PLAYfun	Each task is rated on a 100 mm visual scale with four stepwise increasing boxes (Initial, Emerging, Component, Proficient). The assessor places a mark anywhere along the scale. Each mm is 1 point from 0 to 100 mm Individual ability is measured based on descriptions for each box in the PLAYfun workbook. Points can be summed together to an overall score
		PLAYbasic	Same procedure as PLAYfun
Barnett et al. (2022); PL-C Quest [52]	Physical domain	Physical Literacy in Children Questionnaire (PL-C Quest): pictorial scale with 2 pictures showing opposing actions; 12 items	For each item, the child first determined which of two options is most characteristic of him or her and then decided whether the character in the picture is ‘A lot like me’ or only ‘A bit like me’: four-point scale from 1 to 4
YongKang & QianQian (2022); PLAQ [55]	Physical competence	Physical Literacy self-assessment questionnaire (PLAQ); Self-report questionnaire; 13 items	5-point Likert scale (1: ‘strongly disagree’; 5: ‘strongly agree’)
	Behaviour of physical activity	Physical Literacy self-assessment questionnaire (PLAQ); Self-report questionnaire; 15 items	5-point Likert scale (1: ‘strongly disagree’; 5: ‘strongly agree’)
Stracciolini et al. (2021) [56]	Physical competency	Play, Lifestyle & Activity in Youth Questionnaire; Questionnaire with one section answered by the children and one section answered by the parents about the children; 5% of the items were completely assigned to the physical competency domain; 6% further items crossed over into other domains	Questions with two or multiple answer options; Items were individually assessed
	Daily behaviour	Play, Lifestyle & Activity in Youth Questionnaire; Questionnaire with one section answered by the children and one section answered by the parents about the children; 42% of the items were completely assigned to the daily behaviour domain; 11% further items also crossed over into other domains	Questions with two or multiple answer options; Items were individually assessed
Mota, Martins & Onofre (2021); PPLA [49]	Portuguese Physical Literacy Assessment Observation (PPLA-O) – Movement competence	Teacher-reported proficiency levels in Physical Activities in PE	Teacher selected a level: Introductory proficiency level, elementary proficiency level, advanced proficiency for each physical activity
	Portuguese Physical Literacy Assessment Observation (PPLA-O) – health related fitness	PACER/20-m shuttle run Curl-ups 90º push-ups Backsaver Sit-and-reach Shoulder Stretch	NA
Cairney et al. (2018); Pre PLAy [46]	Movement competences	Pre PLAy tool; 10 items	Early childhood educators allocate children in categories for each item based on free play behaviour and in comparison to children of a similar age. The categories assigned include: ‘does not display skill’, ‘displays skills with instruction’, ‘displays skill without instruction’, ‘displays with other skills’ and ‘creatively displays skills’. Additionally, each item is assessed on a visual scale with 0–15 points
	Coordinated movements	Pre PLAy tool; 4 items	Early childhood educators allocate children on a 4-point adjectival scale from ‘never’ to ‘always’
Belton et al. (2019) [59]	Physical activity	Accelerometers	An ‘optimal’ score for accelerometer-measured PA was defined for this study as an average of 60 min or more of moderate-to-vigorous physical activity (MVPA) daily
		NA [81]	An ‘optimal’ score for habitual PA was defined for this study as meeting the 60-min guideline on 7 days
	Physical competences: fundamental movement skills	The Test of Gross Motor Development-second Edition [82]	For all 12 skills, there were 3–5 specific skill criteria. A fulfilled skill criteria corresponded to one point. A total score of 96 points (48 locomotor, 48 object control) could be gained
	Physical competences: Cardiorespiratory fitness (VO2max)	The Queens College three-minute step test to calculate VO2max [83]	VO2max values were categorised as ‘Very Poor’ (1), ‘Poor’ (2), ‘Fair’ (3), ‘Good’ (4), ‘Excellent’ (5) and ‘Superior’ (6) using the gender-specific normative values for 13–19-year-olds
	Physical competences: Body Mass Index	Stadiometer (Leicester Height Measure) and portable calibrated scales in kilograms	Gender and age-specific cut points were applied to classify BMI into four weight categories: severe thinness (1), thinness (2), overweight (3) and obese (4) [84]
Britton et al. (2022) [60]	Physical competence: object control and locomotor skills	Test of Gross Motor Development-3 [85]	New normative data for age relative to geography, gender, race, ethnicity, household income, and parent education level. Skills and points were the same as in The Test of Gross Motor Development second edition
	Physical competence: balance	The Bruininks–Oseretsky Test of Motor Proficiency—Short Form [86]	Subtest and composite scores can be expressed in age-based standard scores and percentile ranks
	Physical competence: health related fitness	FitnessGram testing battery: shuttle run, back saver, sit and reach, handgrip strength [87]	Calculating a health fitness zone vs need improvement on the FitnessGram official website. Most of the standards have been established to represent a level of fitness associated with some degree of protection against chronic disease
		Isometric plank hold [77]	Time in seconds in isometric plank position; normative score building; max. 10 points
Brown et al. (2020) [61]	Motor competence	The Bruininks–Oseretsky Test of Motor Proficiency—Short Form [86]	Subtest and composite scores can be expressed in age-based standard scores and percentile ranks
Cairney et al. (2019) [62]	Procedure is the same as Brown et al. [61]	Procedure is the same as Brown et al. [61]	Procedure is the same as Brown et al. [61]
Demetriou et al. (2018) [63]	Physical fitness	Several Tests of the German motor performance test DMT 6–18 [88]	Product-based scoring with classification based on normative data
	Fundamental Motor Skills	Several tests of basic motor competence for children in grades one and two; the basic motor competences test [89, 90]	Product-based scoring; a skill can either be passed or failed depending on how many times a skill has been successfully performed. Either 0, 1 or 2 points are awarded per skill. For each subscale, 0–8 points can be gained
	Physical activity behaviour	Self-report question ‘On how many days of last week were you physically active for more than 60 min?’	No description
George et al. (2016) [58]	Motor skills	Movement Assessment Battery for Children [91]	For each child, the raw item scores (e.g. the number of seconds or times a task is performed well) can be transformed into item standard scores, component standard scores, a total standard score and a total percentile score; total standard score for classification: scores at or below the 5^th^ percentile are in the red zone, scores between the 5^th^ and 15^th^ percentile are in the amber zone, scores above the 15^th^ percentile are in the green zone Children who score 15^th^ percentile are classified as children with potential motor problems (at risk or impaired)
	Fitness	Six-minute walk test	Comparison of the distance in metres for pre and post measurement
	Physical activity behaviours	Pedometer	Comparison of steps through the intervention period
Gu et al. (2019) [64]	Fundamental Movement Skills	PE Metrics™ motor skill assessments [92]	Four-point holistic scoring rubric with descriptive qualitative criteria for form and continuity of action at each level. Average scores of the two researchers were calculated to be a motor competency score. Each skill performance scored in Level 3 or above was classified as ‘competent’. Levels 1 and 2 were classified as ‘need improvement’
	Health-related physical fitness	FitnessGram testing battery [87]	Calculating a health fitness zone vs need improvement on the FitnessGram official website. Most of the standards have been established to represent a level of fitness associated with some degree of protection against chronic disease
	School-based MVPA	Accelerometer	Actical accelerometers were adopted to determine MVPA time (light intensity, moderate intensity and vigorous intensity [93]
Guerrero et al. (2018) [65]	Motor competence	Canadian Agility and Movement Skill Assessment [76]	Time and quality of skills are assessed on an obstacle course. Overall, 28 points can be achieved: for time max 14 points based on normative data, for quality max. 14 points in 7 different skills (every skill 1–3 points)
Rudd et al. (2020) [66]	Movement competence: proficiency	Test of Gross Motor Development-3 [85]	New normative data for age relative to geography, gender, race, ethnicity, household income, and parent education level. Skills and points are the same as in The Test of Gross Motor Development second edition
		Test of Stability Skills [94]	NA
	Movement competence: creativity	Divergent Movement Ability Assessment [95]	NA
	Behavioural: physical activity	Accelerometers	Time spent in sedentary, light, moderate and vigorous activity will be determined using age- and population-specific raw acceleration cut-points for the wrist-worn ActiGraph, developed through ongoing research study
Telford et al. (2021) [67]	Physical domain: physical fitness	Accelerometers	MVPA was defined as counts > 2,296 per minute based on previous recommendations [96], using a period of 15 s. The physical activity outcome variables were daily averages of MVPA and total activity counts over 7 days and during school time
	Physical domain: Fundamental Movement Skills	The Test of Gross Motor Development second edition [82]	For 12 skills, there are 3–5 specific skill criteria. A fulfilled skill criteria correspond to one point. A total score of 96 points (48 locomotor, 48 object control) can be gained
Yli-Piipari et al. (2021) [68]	Motor competence	Throwing-catching combination test [97]	Product-based outcome: The final score of the test was the number of correctly performed throwing–catching combinations (out of the 20 attempts)
		Two-leg jump test [98]	Product-based outcome: The final score was the sum of the two attempts
		Balance beam test [98]	Product-based outcome: The final score was the sum of the two attempts
		Five-jump test [98]	Product-based outcome: The final score was the total distance in centimetres of the five jumps
	Health related fitness	The Progressive Aerobic Cardiovascular Endurance Run protocol [87]	The number of lengths completed, including the first length that did not reach the line
		Two tests from the FitnessGram testing battery [87]	Calculating a health fitness zone vs need improvements on the FitnessGram official website. Most of the standards have been established to represent a fitness level associated with some degree of protection against chronic disease
	Physical activity in physical education	Accelerometer	Evenson’s cut points were used to calculate MVPA (≥ 2,296 counts per minute) [96]

*Abbreviations*: *APLQ* Adolescent Physical Literacy Questionnaire, *CAPL* Canadian Assessment of Physical Literacy, *CAPL-2* Canadian Assessment of Physical Literacy second edition, *CAPL 789* Canadian Assessment of Physical Literacy in grades 7–9, *MVPA* Moderate-to-Vigorous Physical Activity, *NA* Not available or data could not be extracted, *PA* Physical activity, *PL* Physical Literacy, *PLAY* Physical Literacy Assessment for Youth, *PLAYbasic* Physical Literacy Assessment for Youth basic, *PLAYfun* Physical Literacy Assessment for Youth fun, *PL-C Quest* Physical Literacy in Children Questionnaire, *PLAQ* physical literacy self-assessment questionnaire, *PPLA* Portuguese Physical Literacy Assessment, *Pre PLAy* Preschool Physical Literacy Assessment Tool

**Table 3:** Assessment tools, procedures and scoring of the affective domain (*n* = 23)

Author(s), year and assessment name	Affective domain: terminology	Affective domain: instrument name	Affective domain: instrument characteristics	Affective domain: assessment procedure
Mohammadzadeh et al. (2021); APLQ [54]	Psychological and behavioural dimension	Adolescent Physical Literacy Questionnaire (APLQ); motivation, self-confidence, attitude, pleasure, and communication, habit of activity, doing outdoor activities	Self-report questionnaire; 11 items	5-point Likert scale (1: ‘Strongly Disagree’; 5: ‘Strongly Agree’). Each item is assigned a maximum of 5 points
Longmuir et al. (2015); CAPL [24]	Motivation and confidence: children’s perception of their ability to be successful in physical activity	The Children’s Self-Perception of Adequacy in and Predilection for Physical Activity Scale [99]	Self-report questionnaire; 9 items of benefits, 10 items barriers subscale	For each item, the child first determined which of two options is most characteristic of him or her and then decided whether it was ‘really true’ or only ‘somewhat true’ of him or her: four-point scale from 1 to 4
	Motivation and confidence: physical activity benefits and barriers	Items were derived from published scales from the Perceived Benefits/Barriers to Exercise Questionnaire [100]	Self-report questionnaire; 9 items benefits, 10 items barriers subscale	5-point Likert scale; Mean and standard deviation of the 5-point Likert scale
Longmuir et al. (2018); CAPL-2 [38]	Motivation and confidence: predilection and adequacy	CAPL-2 questionnaire	Self-report questionnaire; 6 items	For each item, the child first determines which of two options is most characteristic of him or her and then decides whether it is ‘really true’ or only ‘somewhat true’ of him or her: four-point scale from 1 to 4
	Motivation and confidence: perceived competence and intrinsic motivation	CAPL-2 questionnaire	Self-report questionnaire; 6 items	5-point Likert scale (1: ‘Not like me at all”; 5: ‘Really like me’). Each item is assigned a maximum of 2.5 points
Blanchard et al. (2020); CAPL 789 [39]	Motivation and confidence: predilection and adequacy	Proceed in the same way as for CAPL-2	Proceed in the same way as for CAPL-2	Proceed in the same way as for CAPL-2
	Motivation and confidence: perceived competence and intrinsic motivation			
Krenz et al. (2022) [57]	Motivation	German Physical Literacy Assessment for Children – Questionnaire	For children who were six or seven years old the questions were answered in an interview. Older children filled in the questionnaire themselves; 2 items	One item was formulated as open-ended questions. The answers were clustered and rated on a scale of 1 to 4.One Item had a 6-point smiley analogue scale, ranging from the happiest (‘very, very happy’) to the saddest smiley (‘not at all happy’)
	Self-efficacy	German Physical Literacy Assessment for Children – Questionnaire	For children who were six or seven years old the questions were answered in an interview. Older children filled in the questionnaire themselves; Pictorial scale with 2 items	6-point circle analogue scale, ranging from the smallest circle (‘not at all confident’) to the biggest circle smiley (‘fully confident’)
Sum et al. (2018); PPLI [47]	Sense of self and self-confidence	PPLI questionnaire	Self-report questionnaire; 3 items	5-point Likert scale (1: ‘strongly disagree’; 5: ‘strongly agree’); score is computed by adding values of child responses; range from 5 to 15
PLAY-tools (2013) [42]	Confidence in different environments	PLAYself; section environment	Self-report questionnaire; 6 items	5-point scale (never tried, not so good, ok, very good, excellent); Each answer gives zero (never tried), 25 (not so good), 50 (ok), 75 (very good) or 100 (excellent) points. Points can be summed together to an overall PLAYself score
	Self-efficacy	PLAYself; Physical Literacy self-description section	Self-report questionnaire; 12 items	4-point scale (not true at all, not usually true, true, very true); Each answer gives zero, 33, 66 or 100 points. Points can be summed together to an overall PLAYself score
	Self-reported fitness	PLAYself; section fitness	Self-reported question	Two answer options: agree or disagree
	Self-reported importance of movement, activities and sports	PLAYself; item from ‘Relative Ranking of Literacies’ section	Self-reported question	4-point scale (not true at all, not usually true, true, very true); Each answer gives zero, 33, 66 or 100 points. Points can be summed together to an overall PLAYself score
Barnett et al. (2022); PL-C Quest [52]	Psychological domain	Physical Literacy in Children Questionnaire (PL-C Quest)	Pictorial scale with two pictures showing opposing actions; for younger children the text is read out loud; 7 items	For each item, the child first determined which of two options is most characteristic of him or her and then decided whether the character in the picture is ‘A lot like me’ or only ‘A bit like me’: 4-point scale from 1 to 4
YongKang & QianQian (2022); PLAQ [55]	Affective domain	Physical Literacy self-assessment questionnaire (PLAQ)	Self-report questionnaire; 16 items	5-point Likert scale (1: ‘strongly disagree’; 5: ‘strongly agree’)
Stracciolini et al. (2021) [56]	Motivation	Play, Lifestyle & Activity in Youth Questionnaire	Questionnaire with one section answered by the children and one section answered by the parents about the children; 11% of the items were completely assigned to the domain motivation; 14% further items also crossed over into other domains	Questions with two or multiple answer options; Items were individually assessed
Mota, Martins & Onofre (2021); PPLA [49]	Psychological	Portuguese Physical Literacy Assessment Questionnaire	Self-report questionnaire; 46 items	5-point Likert scale (0: ‘Not at all’; 4: ‘totally’)
Cairney et al. (2018); Pre PLAy [46]	Motivation and enjoyment	Pre PLAy tool; 4 items	Early childhood educators rate the child considering children of a similar age; 4 items	5-point Likert scale; score is computed by adding values of early childhood educators’ ratings
Belton et al. (2019) [59]	Psychological correlates – Self-efficacy	Self-efficacy questionnaire [101]	Self-report questionnaire; 10 items	11-point Likert scale (0: ‘not at all confident’; 10: ‘very confident’) Mean and standard deviation of the 11-point Likert scale
	Psychological correlates – Motivation in PE class	Two subscales from the Behavioural Regulations in Physical Education Questionnaire [102]	Self-report questionnaire: both subscales comprised 4 items	5-point Likert scale (1: ‘not true’; 5: ‘very true’) Mean and standard deviation of the 5-point Likert scale
Britton et al. (2022) [60]	Confidence	Physical Activity Self-Efficacy Scale [103]	Self-report questionnaire; one out of three scales; 8 items	3-point Likert scale (1: ‘No’; 2: ‘Not sure’; 3: ‘Yes’) Mean and standard deviation of the 3-point Likert scale
	Motivation	Child-adapted version Behavioural Regulation in Exercise Questionnaire [104]	Self-report questionnaire; 12 items	5-point Likert scale (1: ‘not true for me’; 5: ‘very true for me’) Mean and standard deviation of the 5-point Likert scale
Brown et al. (2020) [61]	Motivation: Predilection and Affect	Two subscales of the Children’s Self-perceptions of Adequacy in and Predilection for Physical Activity [99]	Self-report questionnaire; Predilection subscale 7 items, Enjoyment subscale 3 items	For each item, the child first determined which of two options is most characteristic of him or her and then decided whether it was ‘really true for me’ or only ‘sort of true for me’: 4-point scale from 1 to 4
	Perceived physical competence	Athletic self-concept subscale of Self-Perception Profile for Children [105]	Self-report questionnaire; 6 items	For each item, the child first determined which of two options is most characteristic of him or her and then decided whether it was ‘really true for me’ or only ‘sort of true for me’: 4-point scale from 1 to 4
Cairney et al. (2019) [62]	Motivation – Predilection and Affect	Procedure is the same as Brown et al. [61]	Procedure is the same as Brown et al. [61]	Procedure is the same as Brown et al. [61]
	Perceived physical competence			
Demetriou et al. (2018) [63]	Psychosocial factors – motivation towards physical education	Intrinsic Motivation Inventory [106]	Self-report questionnaire; 9 items	6-point Likert scale (from ‘never’ to ‘always’); mean and standard deviation of the Likert scale
	Psychosocial factors – attitudes towards physical education	An attitude scale for physical education [107]	NA	NA
George et al. (2016) [58]	Awareness/knowledge and understanding	Intrinsic Motivation Inventory [108]	Self-report questionnaire; 21 items	7-point Likert scale (1: ‘strongly disagree’; 7: ‘strongly agree’) Mean and standard deviation of the 7-point Likert scale
		Physical Activity Enjoyment Scale [109]	Self-report questionnaire; 16 items	5-point Likert scale (1: ‘disagree a lot’; 5: ‘agree a lot’); Mean and standard deviation of the 5-point Likert scale
Guerrero et al. (2018) [65]	Motivation	Child-adapted version Behavioural Regulation in Exercise Questionnaire [104]	Self-report questionnaire; 12 items	5-point Likert scale (1: ‘not true for me’; 5: ‘very true for me’) Mean and standard deviation of the 5-point Likert scale
	Confidence	Confidence subscale of the Competitive State Anxiety Inventory-2 for Children [110]	Self-report questionnaire; 5 items	4-point Likert scale (1: ‘not at all’; 4: ‘very much so’); Mean and standard deviation of the 4-point Likert scale
	Perceived physical competence	Measure used previously with children in a physical activity setting [104]	Self-report questionnaire; 6 items	5-point Likert scale (1: not like me at all; 5: really like me); Mean and standard deviation of the 5-point Likert scale
Rudd et al. (2020) [66]	Perceived physical competence	Subscale from The Pictorial Scale of Perceived Competence and Social Acceptance for Young Children [111]	Self-report questionnaire; subscale including 6 items	4-point pictorial scale; score is computed by adding values of child responses; range from 6 to 24
	Perceived skill competence	Pictorial Scale of Perceived Movement Skill Competence for Young Children 3rd Edition [111, 112]	Self-report pictorial questionnaire; 12 items	4-point pictorial scale; score is computed by adding values of child responses; range from 12 to 48
	Self-determined motivation and psychological needs satisfaction	Motivation Assessment Tool for Physical Education [113]	Mixed method tool with quantitative and qualitative elements	Motivation Assessment Tool for Physical Education codebook
Telford et al. (2021) [67]	Psychological domain: physical self-perception	Three subscales of the Children and Youth – Physical Self Perception Profile [114]	Self-report questionnaire; 18 items	For each item, the child first determined which of two options is most characteristic of him or her and then decided whether it was ‘really true’ or only ‘somewhat true’ of him or her: 4-point scale from 1 to 4
	Psychological domain: enjoyment of physical activity	Shortened-Physical Activity Enjoyment Scale [115]	Self-report questionnaire; 7 items	5-point Likert scale (1: ‘disagree a lot’; 5: ‘agree a lot’); Score is computed by adding values of child responses, ranging from 7 to 35; lower scores indicate greater enjoyment
Yli-Piipari et al. (2021) [68]	PE motivation	Finnish version of the Revised Perceived Locus of Causality Scale [116]	Self-report questionnaire; 19 items	5-point Likert scale (1: ‘strongly disagree’; 5: ‘strongly agree’); Mean and standard deviation of the 5-point Likert scale
	PE enjoyment	Finnish version of the enjoyment subscale from the Sport Commitment Questionnaire-2 [117]	Self-report questionnaire; 5 items	5-point Likert scale (1: ‘strongly disagree’; 5: ‘strongly agree’); Mean and standard deviation of the 5-point Likert scale

*Abbreviations*: *APLQ* Adolescent Physical Literacy Questionnaire, *CAPL* Canadian Assessment of Physical Literacy, *CAPL-2* Canadian Assessment of Physical Literacy second edition, *CAPL 789* Canadian Assessment of Physical Literacy in grades 7–9, *IPLA* International Physical Literacy Association, *NA* Not available or data could not be extracted, *PL* Physical Literacy, *PLAY* Physical Literacy Assessment for Youth, *PLAYself* Physical Literacy Assessment for Youth self, *PPLI* Perceived Physical Literacy Instrument, *PL-C Quest* Physical Literacy in Children Questionnaire, *PPLA* Portuguese Physical Literacy Assessment, *PLAQ* physical literacy self-assessment questionnaire, *Pre PLAy* Preschool Physical Literacy Assessment Tool

**Table 4:** Assessment Tools, Procedures and Scoring of the Cognitive Domain (*n* = 14)

Author(s), year and assessment name	Cognitive domain: terminology	Cognitive domain: instrument name	Cognitive domain: instrument characteristics	Cognitive domain: assessment procedure
Mohammadzadeh et al. (2021); APLQ [54]	Knowledge and awareness	Adolescent Physical Literacy Questionnaire (APLQ); knowledge, awareness, and cognition	Self-report questionnaire; 6 items	5-point Likert scale (1: ‘Strongly Disagree’; 5: ‘Strongly Agree’). Each item is assigned a maximum of 5 points
Longmuir et al. (2015); CAPL [24]	Knowledge and understanding	Knowledge and understanding questionnaire	Multiple-choice questionnaire; 10 items	8 out of 10 questions were assessed with 1 point per correct answer; 2 items were assessed with 5 points for a correct answer; maximum score of 18 points
Longmuir et al. (2018); CAPL-2 [38]	Knowledge and understanding	CAPL-2 questionnaire	Multiple-choice questionnaire; 5 items	4 out of 5 questions were assessed using a multiple-choice question format (1 point per correct answer); one question gap text with six words; each correct word is assigned 1 point; maximum score of 10 points
Blanchard et al. (2020); CAPL 789 [39]	Knowledge and understanding	Proceed in the same way as for CAPL-2	Proceed in the same way as for CAPL-2: age-related adjustment	Proceed in the same way as for CAPL-2
Krenz et al. (2022) [57]	Knowledge and understanding	German Physical Literacy Assessment for Children—Questionnaire	For children who were 6 or 7 years old, the questions were answered in an interview. Older children filled in the questionnaire themselves; 3 items	The items were formulated as open-ended questions. The answers were clustered and rated on a scale of 1 to 4
Sum et al. (2018); PPLI [47]	Knowledge and understanding	PPLI questionnaire	Self-reported questionnaire; 3 items	5-point Likert scale (1: ‘strongly disagree’; 5: ‘strongly agree’); cognitive domains score: sum of the item scores (range one to five)
Barnett et al. (2022); PL-C Quest [52]	Cognitive domain	Physical Literacy in Children Questionnaire (PL-C Quest)	Pictorial scale with two pictures showing opposing actions; for younger children, the text is read out loud; 7 items	For each item, the child first determined which of two options is most characteristic of him or her and then decided whether the character in the picture is ‘A lot like me’ or only ‘A bit like me’: four-point scale from 1 to 4
YongKang & QianQian (2022); PLAQ [55]	Knowledge and understanding	Physical Literacy self-assessment questionnaire (PLAQ)	Self-report questionnaire; 15 items	5-point scale (1: ‘strongly disagree’; 5: ‘strongly agree’)
Stracciolini et al. (2021) [56]	Knowledge and understanding	Play, Lifestyle & Activity in Youth Questionnaire	Questionnaire with one section answered by the children and one section answered by the parents about the children; 20% of the items were completely assigned to the domain knowledge and understanding; 13% further items also crossed over into other domains	Questions with two or multiple answer options; Items were individually assessed
Mota, Martins & Onofre (2021); PPLA [49]	Cognitive	Portuguese Physical Literacy Assessment Questionnaire – Knowledge	Multiple-choice questionnaire; 10 items	NA
		Portuguese Physical Literacy Assessment Observation (PPLA-O) – Rules and Tactics	Teacher-reported proficiency levels in Physical Activities in PE	Teacher selected a level: Introductory proficiency level, elementary proficiency level, advanced proficiency for each physical activity
Belton et al. (2019) [59]	Psychological correlates – Perceived benefits of PA	Scale that lists reasons people do PA [118]	Self-reported questionnaire; 9 items	4-point Likert scale (1: ‘very true’; 4: ‘not at all true’); Mean and standard deviation of the 4-point Likert scale
Britton et al. (2022) [60]	Knowledge and understanding	MVPA Guidelines	Multiple-choice question	Score of 1 given for a correct answer and 0 for an incorrect answer
		Sedentary Guidelines	Multiple-choice question	Score of 1 given for a correct answer and 0 for an incorrect answer
Demetriou et al. (2018) [63]	Psychosocial factors – health-related fitness knowledge	Developed questionnaire for a physical education programme ‘HealthyPEP’ [119]	Multiple-choice questionnaire; 9 items	Number of correct answers divided by the total number of questions was calculated to a percentage correct score
Gu et al. (2019) [64]	Health-related fitness knowledge	Standardised written test for third-grade students [120]	Multiple-choice questionnaire; 13 items	Number of correct answers divided by the total number of questions was calculated to a percentage correct score

*Abbreviations*: *APLQ* Adolescent Physical Literacy Questionnaire, *CAPL* Canadian Assessment of Physical Literacy, *CAPL-2* Canadian Assessment of Physical Literacy second edition, *CAPL 789* Canadian Assessment of Physical Literacy in grades 7–9, *PA* Physical activity, *PL* Physical Literacy, *PL-C Quest* Physical Literacy in Children Questionnaire, *PLAQ* physical literacy self-assessment questionnaire, *PPLA* Portuguese Physical Literacy Assessment, *MVPA* Moderate-to-Vigorous Physical Activity

### Consideration of the PL domains

All three PL domains were addressed in 12 assessment tools [24, 38, 39, 49, 52, 54–57, 59, 60, 63] (see Table 5). The physical domain was collected in 22 assessment procedures; only the PPLI did not include this domain [47]. Reasons for omission were not given.

**Table 5:** Assessing physical literacy as a holistic approach: procedure of the different research groups

Author(s), year and assessment name	Cognitive domain	Affective domain	Physical domain	Unassignable additional domain	Assessing physical literacy as holistic approach; scoring system
Mohammadzadeh et al. (2021); APLQ [54]	✓	✓	✓	NA	Every item score (a ranking from 1 to 5 on the Likert scale) was summed together to one final PL score (ranging from 25 to 125)
Longmuir et al. (2015); CAPL [24]	✓	✓	✓	NA	Additive score formation of maximum 100 points; knowledge and understanding 18 points, motivation and confidence 18 points, physical competence 32 points, daily behaviour 32 points The scores for each protocol, domain scores, and the overall CAPL score continue to be interpreted within 4 categories based on normative data for age and gender, as follows: Beginning = less than the 17^th^ percentile Progressing = 17^th^ to 65^th^ percentiles Achieving = above the 65^th^ percentile to the 85^th^ percentile Excelling = above the 85^th^ percentile
Longmuir et al. (2018); CAPL-2 [38]	✓	✓	✓	NA	Additive score formation of maximum 100 points; cognition 10 points, motivation 30 points, physical 30 points, habitual engagement 30 points The scores for each protocol, domain scores, and the overall CAPL-2 score continue to be interpreted within 4 categories based on normative data for age and gender, as follows: Beginning = less than the 17^th^ percentile Progressing = 17^th^ to 65^th^ percentiles Achieving = above the 65^th^ percentile to the 85^th^ percentile Excelling = above the 85^th^ percentile
Blanchard et al. (2020); CAPL 789 [39]	✓	✓	✓	NA	Procedure is the same as CAPL-2
Krenz et al. (2022) [57]	✓	✓	✓	NA	Additive score formation of maximum 60 points; knowledge and understanding 6 points, motivation, and self-efficacy 18 points, motor skills 18 points, participation 18 points
Sum et al. (2018); PPLI [47]	✓	✓	×	Self-expression and communication with others; 3-item questionnaire; 5-point Likert scale (1: strongly disagree; 5: strongly agree)	Every item score (a ranking from 1 to 5 on the Likert scale) was summed together to a final PL score ranging from 9 to 45
PLAY tools (2013) [42]	×	✓	✓	PLAYself self-report questionnaire; self-report question about the importance of maths, writing and reading	Domains were individually assessed
Barnett et al. (2022); PL-C Quest [52]	✓	✓	✓	Social domain; 4 items; proceeds in the same way as the other PL-C Quest scales	All item scores (rankings from 1 to 4) were summed to obtain a final PL score ranging from 30 to 120
YongKang & QianQian (2022); PLAQ [55]	✓	✓	✓	NA	Domains were individually assessed
Stracciolini et al. (2021) [56]	✓	✓	✓	NA	Items were individually assessed
Mota, Martins & Onofre (2021); PPLA [49]	✓	✓	✓	Social domain; 43 items; proceeds in the same way as the other PPLA-Q scales	Domains were individually assessed
Cairney et al. (2018); Pre PLAy [46]	×	✓	✓	NA	Two approaches were taken in the scoring system: Overall, PL was rated by one item from 0 to 15 points on a visual scale from Early Childhood Educators. Pre PLAy overall score was made by summing all item scores (ten items in movement competence, four items in coordinated movements, four items in motivation and enjoyment)
Belton et al. (2019) [59]	✓	✓	✓	NA	Domains were individually assessed
Britton et al. (2022) [60]	✓	✓	✓	NA	Domains were individually assessed
Brown et al. (2020) [61]	×	✓	✓	NA	Building 5 different PL profiles characterised by varying combinations of scores across the domains: Profile 1: Children in this profile were defined by consistently low scores for motor competence, confidence and motivation, along with a very low enjoyment score Profile 2: Children in this profile were distinguished by consistently low scores across each of the domains of PL Profile 3: These children were distinguished by a high score for enjoyment and consistently low scores for motor competence, confidence and motivation Profile 4: These children were characterised by consistently moderate scores across each of the domains of PL Profile 5: Children in this profile were defined by consistently high scores across each of the domains of PL
Cairney et al. (2019) [62]	×	✓	✓	NA	Domains were individually assessed
Demetriou et al. (2018) [63]	✓	✓	✓	Cognitive performance; Simon task in the form of a Dots test and the Flanker test [121, 122]	Domains were individually assessed
George et al. (2016) [58]	×	✓	✓	Rosenberg Self-Esteem Scale; 10 items questionnaire; 5-point Likert scale [123]	Domains were individually assessed
Gu et al. (2019) [64]	✓	×	✓	NA	Domains were individually assessed
Guerrero et al. (2018) [65]	×	✓	✓	NA	Domains were individually assessed
Rudd et al. (2020) [66]	×	✓	✓	Executive function National Institute for Health Toolbox to assess the three core executive functions [124] Self-regulation Strength and Difficulties Questionnaire [125, 126]	Domains were individually assessed
Telford et al. (2021) [67]	×	✓	✓	NA	Domains were individually assessed
Yli-Piipari et al. (2021) [68]	×	✓	✓	NA	Domains were individually assessed

*Abbreviations*: *APLQ* Adolescent Physical Literacy Questionnaire, *CAPL* Canadian Assessment of Physical Literacy, *CAPL-2* Canadian Assessment of Physical Literacy second edition, *CAPL 789* Canadian Assessment of Physical Literacy in grades 7–9, *NA* Not available or data could not be extracted, *PA* Physical activity, *PL* Physical Literacy, *PPLI* Perceived Physical Literacy Instrument, *PLAY* Physical Literacy Assessment for Youth, *PLAYself* Physical Literacy Assessment for Youth self, *PL-C Quest* Physical Literacy in Children Questionnaire, *PLAQ* physical literacy self-assessment questionnaire, *PPLA* Portuguese Physical Literacy Assessment, *PPLA-O* Portuguese Physical Literacy Assessment—Observation*, Pre PLAy* Preschool Physical Literacy Assessment Tool

The affective domain was also included in 22 of the 23 assessment procedures. Gu et al.’s [64] combined this domain with the cognitive domain in their PL definition and did not explicitly operationalise the affective PL domain.

The cognitive domain was considered in 14 assessment systems [24, 38, 39, 47, 49, 52, 54–57, 59, 60, 63, 64].

Only in eight of the 23 systems analysed, was PL assessed in the form of a total score resulting from the domain scores [24, 38, 39, 46, 47, 52, 54, 57]. In CAPL-2 and CAPL 789, the total score and the domain scores were additionally classified in an age- and gender-adjusted percentile system [38, 39]. On this basis, children can be assigned to one of four categories: Beginning, Progressing, Achieving and Excelling. Brown et al. [61], in contrast, designed an individual profile based on the domain characteristics (see Table 5).

### Evaluation of the assessment systems

Varying statistical procedures were used to validate the scoring systems. For some of the PLAY tools [42] as well as the CAPL [24], German Physical Literacy Assessment for Children [57], PLAQ [55], APLQ [54], and PPLI [47] exploratory factor analysis was conducted to generate the factor structure from empirical data, exclude individual items, check the internal consistency of the scales and thus address construct validity. In addition, confirmatory factor analysis was conducted to assess construct validity [24–26, 28, 29, 35, 36, 44, 47, 52, 54, 55, 60]. Longmuir et al. [24] employed correlational analysis between children’s CAPL domain scores and teachers’ assessments of children’s PL to determine convergent validity. The PLAY tools were also tested against a variety of instruments to examine convergent validity: the PLAYfun and PLAYbasic scores were compared with other motor test scores [43], and physical activity was measured using pedometers [45] or questionnaires [40]. The PLAYself questionnaire from the PLAY tools and the PPLA-O tool were validated based on item response theory and tested for the unidimensionality of the scales [41, 50]. The PPLA-Q was checked for content validity using the Mokken scale analysis [51].

## Discussion

PL is becoming increasingly important as a potential guiding concept for the promotion of physical activity and health due to its holistic approach. However, there is currently neither a uniform framework concept nor a corresponding assessment procedure established in Germany to develop and test appropriate interventions. A scoping review was thus conducted to summarise current definitions and test methods, including assessments of individual PL, for children and adolescents.

A total of 23 models and procedures were identified, some of which varied considerably. As previous reviews have also shown, the assessment procedure developed depended on the underlying definition of PL and the context in which the procedure was used [20, 21].

Only 12 assessment instruments addressed all core components of the Whiteheadian conceptualisation (see Table 5). In the CAPL, CAPL-2, CAPL 789, PPLI, APLQ, PL-C Quest, German Physical Literacy Assessment for Children and Pre PLAy, a total score was determined by summing individual domain scores. Although a sum score can be used to quantify as well as compare PL status within and between groups, a more nuanced and individualised consideration of strengths or weaknesses may be more meaningful in terms of PL promotion. A promising approach is the profiling performed by Brown et al. [61]. Each child was assigned to one of five profiles based on their manifestation within the domains. For example, children with low scores in ‘motor competence’ and ‘confidence and motivation’ and a very low score in ‘enjoyment’ were assigned to profile 1, termed ‘inconsistently low PL’. Profile 3, on the other hand, was characterised by low scores in ‘motor competence’ and ‘confidence and motivation’ and a high score in ‘enjoyment’. The researchers showed that children with a ‘better’ PL profile were more physically active. This approach appears to align more closely with the holistic perspective on the individual in the Whiteheadian PL tradition [17].

One domain – the physical domain – was predominantly integrated into the reviewed systems’ consideration of PL, consistent with the findings of earlier reviews [20, 22]. Only one approach did not assess the physical domain as physical or motor skills [47]. Jean de Dieu and Zhou showed that 70% of the PL assessment systems they identified only addressed the physical domain [22]. Whitehead criticised this rather one-sided view of physical activity and sport as insufficient for developing an active lifestyle [18], as it does not do justice to the holistic view of physical activity behaviour. Although simplification of certain aspects of the theory may be necessary due to the operationalisation process, assessment systems should always maintain the theoretical model as their basis [127]. The affective domain was also identified in almost all assessment procedures found, with only one instrument not considering this domain [64]. In most cases, existing questionnaires were employed to assess, for example, intrinsic motivation (see Table 3).

The cognitive domain (i.e. following the Whiteheadian definition knowledge and understanding of changes in the body and mind through exercise [17]) was only considered in 14 of the assessment procedures. However, the status of the research has improved considerably over time in this area in particular. While in the initial search in July 2022, only seven out of 16 identified assessment systems addressed this area, by May 2023, 14 of the 23 identified systems included this domain. The assessment tools utilised questionnaires or teacher assessments to capture the cognitive domain. In the case of questionnaires, alongside self-report questionnaires, multiple-choice questionnaires were frequently employed to represent children’s knowledge [24, 38, 39, 49, 60, 63, 64]. In assessment systems that did not consider the cognitive domain, the reasons for its exclusion were manifold. In some cases, it was not included in the definition of PL [68], and in other, corresponding data were not collected [61, 62]. In Rudd et al. [66] and George et al. [58], the cognitive domain was described in the assessment but did not match the underlying understanding of the cognitive domain adopted in this work. While we were guided by Whitehead’s definition, which emphasis knowledge and understanding of physical and psychological changes due to exercise, George et al. [58] focused on intrinsic motivation (affective domain) and Rudd et al. [68] focused on executive functions and self-regulation. The remaining research groups did not justify the omission of the cognitive domain [42, 65, 67].

Presumably, this inconsistency in measuring the cognitive components of PL is due to the challenge of systematically capturing and, most importantly, to defining the content that should be addressed in this domain. There is currently no consensus on what knowledge regarding the effects of sport and exercise can be expected for children and adolescents at different age levels. The CAPL, for instance, is oriented towards the contents of the Canadian school sports curriculum [24]. However, this is not transferable to other geographical regions and particular to other age groups, such as pre-school aged children. Cairney et al. [46] justified the absence of the cognitive domain from the Pre PLAy tool by arguing that it was not developmentally suitable for preschool-aged children. Of greater importance at this early age appears to be a specific attitude towards movement or an understanding or feeling of what movement entails. Such a focus would likely align with the original PL approach, but quantitatively capturing it could prove difficult.

### Strengths and limitations

This study was conducted based on a literature search of three databases: SPORTDiscus, PubMed and Web of Science. This approach covered a broad range of research in sports science and sports medicine, but educational literature was underrepresented. The databases used were chosen because of the health- and prevention-oriented focus of the scoping review.

Only papers written in English were included in this work. The literature search did identify several articles from Asia that were not included due to the language of the text. Therefore, it cannot be ruled out that relevant literature may have been overlooked.

During our publication process, a rapid increase in literature and assessment systems in the field of PL was observed. Therefore, a follow-up research was conducted, but it can be assumed that the research area is still expanding, further relevant and innovative developments will occur.

Finally, it is possible that the requirement that at least two of the three domains be addressed in the PL assessment also led to a reduction in possible search results. However, we chose this approach to do justice to the holistic nature of the PL concept.

## Conclusion

The PL construct is a promising concept for promoting physical activity in childhood and adolescence because it considers multiple facets of physical activity and is thus intended to lay an essential foundation for an active lifestyle throughout the lifespan. However, to enable the development of concrete promotion measures based on this holistic approach, a clear and theory-based definition and assessment derived from it are needed. Unfortunately, the holistic nature of the approach, though promising, is precisely what makes adequate assessment a methodological challenge due to its complexity. In addition, the developmental stages of children and adolescents must be taken into account. A certain abstraction from the original construct may be unavoidable when putting the theory into practice, but the fundamental PL approach should always remain in focus. Priority should therefore be given to developing assessments that are holistic instruments rather than merely domain-specific constructs.

## Data Availability

All data generated or analysed during this study are included in this published article.

## Abbreviations

APLQ: Adolescent Physical Literacy Questionnaire
BMI: Body mass index
CAPL: Canadian Assessment of Physical Literacy
CAPL-2: Canadian Assessment of Physical Literacy second edition
CAPL 789: Canadian Assessment of Physical Literacy in grades 7–9
IPLA: International Physical Literacy Association
MVPA: Moderate-to-vigorous physical activity
NA: Not available
PA: Physical activity
PL: Physical literacy
PLAY: Physical Literacy Assessment for Youth
PLAYbasic: Physical Literacy Assessment for Youth basic
PLAYfun: Physical Literacy Assessment for Youth fun
PLAYself: Physical Literacy Assessment for Youth self
PLAQ: Physical Literacy Self-Assessment Questionnaire
PL-C Quest: Physical Literacy in Children Questionnaire
PPLA: Portuguese Physical Literacy Assessment
PPLA-O: Portuguese Physical Literacy Assessment-Observation
PPLA-Q: Portuguese Physical Literacy Assessment-Questionnaire
PPLI: Perceived Physical Literacy Instrument
Pre PLAy: Preschool Physical Literacy Assessment Tool
PRISMA: Preferred Reporting Items for Systematic Reviews and Meta-Analyses
